# Efficacy and Safety of Yangyin Shuxin Decoction—a Chinese Herbal Medicine Formula For Heart Failure with Preserved Ejection Fraction: A Randomized Controlled Trial

**DOI:** 10.1155/2022/1486366

**Published:** 2022-08-22

**Authors:** Qing Li, Lishuo Su, Ruijuan Zhou, E. Tang, Yu Liu, Tao Cheng, Shuai Wang, Zhiqiang Zhao, Xianliang Wang, Jingyuan Mao

**Affiliations:** ^1^Department of Cardiovascular Diseases, First Teaching Hospital of Tianjin University of Traditional Chinese Medicine, National Clinical Research Center for Chinese Medicine Acupuncture and Moxibustion, Tianjin, China; ^2^Tianjin University of Traditional Chinese Medicine, Tianjin, China

## Abstract

**Background:**

Heart failure with preserved ejection fraction (HFpEF) is a large subtype of heart failure (HF) characterized by exercise intolerance and reduced quality of life. Studies have shown that traditional Chinese medicine (TCM) combined with conventional Western medicine has a good effect on improving exercise tolerance and quality of life in patients with HFpEF, but the overall quality of evidence is low. This study aimed to determine the safety and efficacy of Yangyin Shuxin (YYSX) decoction in the treatment of HFpEF.

**Methods:**

A prospective, single-blind, single-center, randomized controlled clinical study was conducted. 64 patients with HFpEF were randomly assigned to receive oral YYSX decoction (150 ml, twice a day) combined with conventional Western treatment or conventional Western treatment alone at a ratio of 1 :  1. The course of treatment was 2 weeks, and the follow-up was 3 months. The primary outcome was peak oxygen uptake (peak VO_2_) measured by the cardiopulmonary exercise test (CPET). Furthermore, the safety of YYSX decoction was assessed.

**Results:**

63 patients (31 in the YYSX group and 32 in the control group) were included in the full analysis set. The peak VO_2_ of the YYSX group was significantly higher than that of the control group (12.04 ± 3.41 vs. 11.02 ± 3.33, *P* = 0.013) after 2 weeks. The maximum voluntary ventilation (MVV) was significantly higher in the YYSX group compared with the control group (*P* < 0.05). The YYSX group had a higher EQ-visual analogue scale (EQ-VAS) score (71.13 ± 13.95 vs. 70.94 ± 13.70, *P* < 0.05) and a lower TCM Four-Dimensional Diagnostic Information Scale (TCMFDIS) score (49.74 ± 24.73 vs. 64.16 ± 27.15, *P* < 0.05) than the control group. There was no statistical difference between two groups (*P* = 0.160), although 51.61% of patients in the YYSX group showed a decrease in brain natriuretic peptide (BNP) levels of at least 30%, compared with 37.50% of patients in the control group. No serious adverse events were reported in either group, but systolic and diastolic blood pressure decreased and serum sodium levels increased slightly in the control group.

**Conclusion:**

The YYSX decoction combined with conventional Western treatment was superior to the conventional Western treatment alone in improving exercise tolerance, quality of life, and cardiopulmonary function of patients with HFpEF. YYSX decoction is safe and may prevent a drop in blood pressure and sodium retention. Trial Registration. Chinese Clinical Trial Registry (www.chictr.org/cn/, No. ChiCTR-IOR-17014206).

## 1. Introduction

Heart failure with preserved ejection fraction (HFpEF) accounts for 40–55% of all heart failure cases, with a rising prevalence over time and a mortality rate equivalent to heart failure with reduced ejection fraction (HFrEF) [1]. HFpEF patients are characterized by exercise intolerance and reduced quality of life [2]. There is a need for treatments that minimize the symptoms and improve the exercise tolerance and quality of life of patients with HFpEF, while reducing hospitalization rates and extending life span [3].

Studies have shown that traditional Chinese medicine (TCM) combined with conventional Western medicine has a good effect on improving exercise tolerance and quality of life in patients with HFpEF, but there are still some shortcomings that limit the reliability and extrapolation of results. Further studies with well-defined patient population, clear inclusion and exclusion criteria, as well as multidimensional endpoints are required [4]. Based on TCM theory and our decades of experience, we held the opinion that yin deficiency, blood stasis, and phlegm-heat are the fundamental causes of HFpEF, and hence YYSX decoction was developed for therapeutic use in the treatment of HFpEF. Yangyin Shuxin (YYSX) decoction is a Chinese herbal medicine formula composed of 10 Chinese herbs, including Shanzhuyu (*Cornus officinalis Sieb et Zucc*), Maidong (*Ophiopogon japonicus (Thunb.) Ker-Gawl*), Huangjing (*Polygonatum kingianum Collett et Hemsl*), Huanglian (*Coptis chinensis Franch*), Biejia (*Trionyx sinensis Wiegmann*), Danshen (*Salvia miltiorrhiza Bunge*), Dilong (*Pheretima aspergillum (E.Perrier)*), Banxia (*Pinellia ternata (Thunb.) Makino*), Gualoupi (*Trichosanthes kirilowii Maxim*), and Zhiqiao (*Citrus* × *aurantium L*), which has the effects of nourishing yin (*Yangyin*), promoting blood circulation (*Huoxue*), resolving phlegm (*Huatan*), and clearing away heat (*Qingre*). The good effect of YYSX decoction in patients with HFpEF has been clinically verified [5]. Previously, we conducted a preliminary assessment of the efficacy of YYSX decoction in improving the quality of life in patients with HFpEF, using Minnesota Living with Heart Failure Questionnaire (MLHFQ) as the primary endpoint (Chinese Clinical Trial Registry, No. ChiCTR-IIR-15007620) [6]. It has shown certain advantages of lowering the MLHFQ score and relieving symptoms of patients with HFpEF. In addition, we have applied for patent protection for YYSX decoction (patent number ZL201610782255.7, see Additional file S1). In this study, we aimed to use more objective indicators to further evaluate its efficacy and safety.

In this study, an incremental cardiopulmonary exercise test (CPET) with gas exchange measurement was used to evaluate the peak oxygen consumption (peak VO_2_), which is the gold standard assessment of aerobic capacity. However, the CPET has not been widely used in clinical research because of its large data collection, difficult analysis, and the expense of equipment in the past. It has been proved that peak VO2, oxygen uptake at anaerobic threshold (VO2@AT), and the minute ventilation/carbon dioxide production (VE/VCO2) slope have prognostic value for patients with HFrEF. Importantly, recent studies have shown that the degree of diastolic dysfunction and prognosis of patients with HFpEF are also closely related to the above indicators of CPET [7–9].

Therefore, we selected VO_2_ peak as the primary outcome and combined with other CPET indicators, echocardiographic measures, brain natriuretic peptide (BNP) level, pulmonary function test (PFT) parameters, European Quality of Life Five-Dimension Five-Level Scale (EQ-5D-5 L), and TCM Four-Dimensional Diagnostic Information Scale (TCMFDIS) to objectively, accurately, and comprehensively evaluate the efficacy and safety of YYSX decoction in enhancing exercise capacity in patients with HFpEF.

## 2. Materials and Methods

### 2.1. Study Design

A prospective, single-blind, single-center, parallel randomized controlled trial (RCT) was conducted in the First Teaching Hospital of Tianjin University of Traditional Chinese Medicine. The research protocol was approved by the Research Ethics Committee of the First Teaching Hospital of Tianjin University of Traditional Chinese Medicine (No. TYLL2017[K]034) and followed the Declaration of Helsinki's ethical guidelines. It was registered at the Chinese Clinical Trial Registry (Registration number: ChiCTR-IOR-17014206). 64 participants provided written informed permission after receiving a thorough overview of the research methods and were informed that the study's data will be published anonymously in scientific journals. The findings of this study were reported using the Consolidated Standards of Reporting Trials standards (CONSORT checklist, see Table S2).

According to the coding sequence from a preset random number table, patients were randomly assigned to the treatment or control group in a 1 : 1 ratio. The compound decoction was not disguised, and no placebo was utilized. Each group received either oral YYSX decoction (150 ml, twice a day) combined with conventional Western treatment and conventional Western treatment alone for 2 weeks. After the completion of 2 weeks' treatment, there were three follow-up evaluations at 4, 8, and 12 weeks, as detailed in the design paper [10].

Each researcher received training on the specifics of filling out study forms, as well as associated operating processes and data management. A data monitoring committee was formed, primarily to conduct interim analyses and evaluate adverse occurrences. The committee addressed any changes to the primary research protocol as well as the key trial processes and materials. Any adverse events were documented in the CRF and promptly reported to the data monitoring committee.

### 2.2. Patients

We recruited 64 patients with HFpEF between January 2018 and September 2020. The *2016 ESC Guidelines for the Diagnosis and Treatment of Acute and Chronic Heart Failure* were used as the reference diagnostic criteria for HFpEF. The diagnosis should include the following: (1) the presence of symptoms such as breathless, orthopnoea, paroxysmal nocturnal dyspnoea, reduced exercise tolerance, and/or signs such as elevated jugular venous pressure, hepatojugular reflux, third heart sound (gallop rhythm), later displaced apical impulse of HF; (2) LVEF ≥50; (3) BNP >35 pg/ml; and (4) meet at least one of the following criteria: (1) objective demonstration of structural alterations of the heart: left atrial volume index (LAVI) >34 ml/m^2^ or a left ventricular mass index (LVMI) ≥115 g/m^2^ for males and ≥95 g/m^2^ for females; (2) objective demonstration of functional alterations of the heart: E/e' ≥13 and a mean e' septal and lateral wall <9 cm/s.

Participants who met the diagnostic criteria, were older than 40 years, and agreed to provide written informed consent were included in this study. Participants who satisfied one or more of the following criteria were not allowed to participate: (1) with acute coronary syndrome, hypertrophic cardiomyocyte, and restrictive cardiomyopathy; (2) with a recent history of intracoronary stent implantation, permanent pacemaker, implantable defibrillator (ICD), or left ventricular assist device implantation or coronary artery bypass grafting within 90 days; (3) systolic pressure >160 mmHg or <90 mmHg at intake; (4) chronic lung illness needing oxygen or drug therapy; (5) cerebral infarction within 90 days; (6) patients with severe liver, renal failure, malignant tumor, or other comorbidities needing special treatments, which may affect the clinical management of HFpEF; (7) pregnant or breastfeeding women or women of childbearing potential who are not using reliable contraception; (8) inability to perform a cardiopulmonary exercise test; (9) with poor compliance; and (10) participated in other studies within 2 months.

### 2.3. Intervention and Comparator

Eligible patients were randomized to receive conventional treatment alone or the YYSX decoction (150 ml, twice a day) combined with conventional Western treatment. The conventional treatment is implemented based on the *China Heart Failure Diagnosis and Treatment Guide 2014* and *2016 ESC Guidelines for the Diagnosis and Treatment of Acute and Chronic Heart Failure*. Routine treatment generally includes the following:

1. Blood-pressure control: the target blood pressure should be lower than 130/80 mmHg, which is the standard for patients with simple hypertension.
2. Application of diuretics: eliminating fluid retention and edema can relieve pulmonary stasis and improve cardiac function. However, excessive diuresis is not recommended to avoid hypotension caused by excessive preload reduction.
3. Control and treatment of other underlying diseases and comorbidities: control of ventricular rate in chronic atrial fibrillation with non-dihydropyridine calcium channel blockers (diltiazem or verapamil) or beta-blockers, if possible, to convert and maintain sinus rhythm, is beneficial to patients. Aggressive treatment of diabetes mellitus and glycemic control and reduce weight in obese individuals should be recommended. For patients with left ventricular hypertrophy, ACEI, ARB, and β-blocks can be used to reverse left ventricular hypertrophy and improve left ventricular diastolic function of left ventricle (LV) hypertrophy and improvement in LV diastolic function with ACEI, ARB, and *β*-blockers in patients with LV hypertrophy. Digoxin does not increase myocardial relaxation and is not recommended.
4. Health education: help patients follow a healthy lifestyle (eg, salt and water restriction, weight monitoring, exercise, and quitting smoking and drinking alcohol), therapeutic medications, and emergency management instructions.

The composition of YYSX decoction is *Cornus officinalis Sieb et Zucc* 15 g, *Ophiopogon japonicus (Thunb.) Ker-Gawl* 20 g, *Polygonatum kingianum Collett et Hemsl* 15 g, *Coptis chinensis Franch* 10 g, *Trionyx sinensis Wiegmann* 10 g, *Salvia miltiorrhiza Bunge* 20 g, *Pheretima aspergillum (*E. *Perrier)* 10 g, *Pinellia ternata (Thunb.) Makino* 10 g, *Trichosanthes kirilowii Maxim* 15 g, and *Citrus* × *aurantium L* 12 g. Prepared slices of the Chinese crude drugs were all obtained from the herbal pharmacy of the First Teaching Hospital of Tianjin University of TCM. The same herbs are sourced from the same place of origin. The YYSX decoction is prepared by a uniform decoction in the herbal pharmacy in accordance with the Management Standards of Chinese Medicine Decoction Room in Medical Institutions (2009).

In addition, all participants were not allowed to use other TCM preparations within 2 weeks before participating the study.

### 2.4. Outcome and Assessment

The primary endpoint was peak VO_2_ tested using the CPET. Secondary endpoints consisted of VO_2_@AT and VE/VCO_2_ slope detected in the CPET, the ratio between early diastolic transmitral flow and mitral annular velocity (E/e'), left atrial volume index (LAVI), left ventricular mass index (LVMI), isovolumic relaxation time (IVRT), and mitral E deceleration time (EDT) detected with ultrasonic cardiogram, BNP, maximum voluntary ventilation (MVV), forced expiratory volume in 1 second (FEV1), forced vital capacity (FVC), and inspiratory capacity (IC) examined by PFT, EQ-5D-5 L, TCMFDIS score, compound endpoint events, and so on.

Security outcomes were assessed based on reports of adverse events, physical examination, vital signs, and laboratory tests. During the intervention, all participants were asked to report any negative experiences. Weight and height of the individuals were measured before and after a two-week period. Each visit included a check of systolic blood pressure (SBP), diastolic blood pressure (DBP), and heart rate (HR). Laboratory tests included complete blood count (white blood cell (WBC), red blood cell (RBC), hemoglobin (HGB), and platelets (PLT)) and blood chemistry (alanine aminotransferase (ALT), creatinine (Cr), blood urea nitrogen (BUN), and Na^+^, K^+^, Cl^−^ concentrations).

### 2.5. Sample Size and Statistical Analysis

The sample size of this study was determined based on the assumption that the peak VO_2_ on day 7 in the treatment group was 3.0 ± 3.6 ml/kg/min and the peak VO_2_ on day 7 in the test group was 0.4 ± 2.7 ml/kg/min [11]. The treatment and control groups had a sample size distribution of 1 : 1. As a result, a total of 64 individuals (32 per group) were chosen using a statistical power (1−*β*) of 90% and a significance threshold of 5% (one-sided test), allowing for a dropout percentage of 20%.

Data are expressed as mean ± standard deviation (SD) for normally distributed data and as median (interquartile range (IQR)) for non-normally distributed data. For normally distributed data, the between-group differences were analyzed using ANCOVA, with intervention condition as the group factor and preintervention value as the covariate. The intragroup differences before and after treatment were analyzed using the paired *T*-test. For non-normally distributed data, the Wilcoxon rank sum (Mann–Whitney U) test was used. Repeated measurements of paired data were analyzed using the Friedman test with Dunn's multiple comparisons test (non-normal distribution).

## 3. Results

### 3.1. Patient Disposition

**Figure 1:**
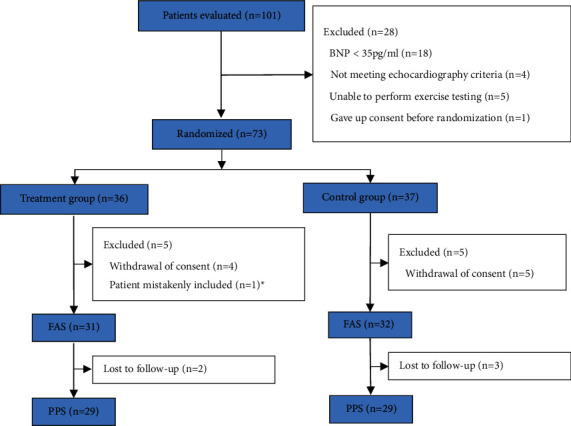
Participant flow chart. ^*∗*^One patient was mistakenly randomized into the trial due to human error, and therefore, we exclude the patient's data from analysis.

A total of 101 patients were assessed for eligibility; 73 patients with HFpEF met the inclusion criteria were randomized 1 : 1 into two groups (36 in the treatment group and 37 in the control group). Among the enrolled participants, 9 were withdrawn during the treatment phase of the study upon patients' request. 1 patient was inappropriately randomized into the clinical trials before the echocardiography as a result of human error. Trial investigators excluded this patient's data from analysis according to the literature [12], without risk of bias. Therefore, 63 patients were included in the FAS (31 in the treatment group and 32 in the control group) and 58 patients were included in the PPS (29 in each group).  Figure 1 depicts the patient disposition as well as the distribution of the research groups.

### 3.2. Baseline Characteristics

Table 1 shows the baseline demographic and clinical characteristics of the patients in the treatment and control groups. Our data showed that patients with HFpEF tend to be female and older, which is consistent with previous studies. The mean age was 69.44 ± 6.04 years, and 79.37% were women. Both groups were comparable in terms of the demographic characteristics, past medical history, NYHA functional classification, and concomitant medications (*P* < 0.05).

SBP: systolic blood pressure; DBP: diastolic blood pressure; HR: heart rate; HTN: hypertension; DM: diabetes mellitus; HLD: hyperlipidemia; CHD: coronary heart disease; NYHA: New York Heart Association; ACEI: angiotensin-converting enzyme inhibitors; ARB: angiotensin receptor blockers; CCB: calcium channel blocker; MRA: mineralocorticoid receptor antagonist.

### 3.3. Efficacy

#### 3.3.1. Primary Outcome

**Figure 2:**
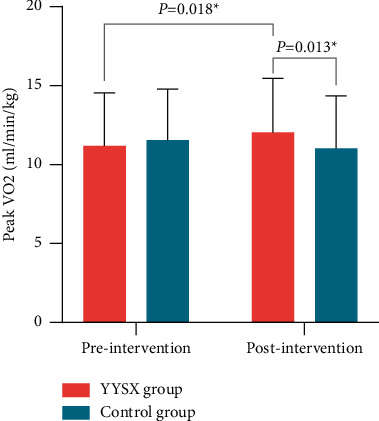
Comparison of peak oxygen uptake (peak VO_2_) in treatment and control groups between baseline and 2 weeks.

There was a substantial difference in peak VO_2_ between the YYSX and control groups after 2 weeks of treatment (Table 2). The peak VO_2_ was significantly higher than the control group (12.04 ± 3.41 vs. 11.02 ± 3.33, *P*=0.013), suggesting that YYSX decoction combined with conventional Western treatment was more effective than conventional Western treatment regarding the peak VO_2_. Moreover, the peak VO_2_ in the YYSX group after 2 weeks was significantly higher than baseline (*P*=0.018), whereas in the control group it was significantly lower than the baseline (Figure 2).

#### 3.3.2. Secondary Outcome

For the VO_2_@AT and VE/VCO_2_ slope in the CPET, there was no significant difference between the treatment and control groups. In intragroup comparison, an upward trend in VO_2_@AT was observed compared to the baseline in the YYSX group, but there is little difference (*P* < 0.05).

**Figure 3:**
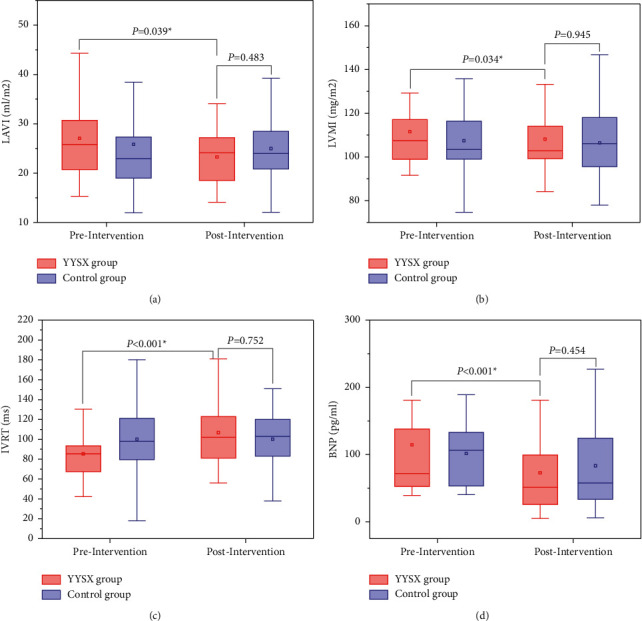
The effect of YYSX decoction in cardiac function: a. comparison of left atrial volume index (LAVI) in treatment and control groups between baseline and 2 weeks; b. comparison of left ventricular mass index (LVMI) in treatment and control groups between baseline and 2 weeks; c. comparison of isovolumic relaxation time (IVRT) in treatment and control groups between baseline and 2 weeks; and d. comparison of brain natriuretic peptide (BNP) in treatment and control groups between baseline and 2 weeks.

As shown in Table 2, among the indicators tested by cardiac ultrasound, LAVI, LVMI, and IVRT significantly increased in the treatment group compared to the baseline (*P* < 0.05). However, there was no significant difference between the two groups regarding LAVI, LVMI, and IVRT. No significant difference in E/e' and EDT was observed in comparisons within and between groups (Figure 3).

After 2 weeks of treatment, BNP levels were significantly lower in both groups from baseline but were significantly lower in the YYSX group than in the control group. (*P* < 0.001). Moreover, BNP was reduced by at least 30% in 51.61% of patients in the YYSX group and 37.50% in the control group, but the difference was not statistically significant (*P*=0.160) (Figure 3).

**Figure 4:**
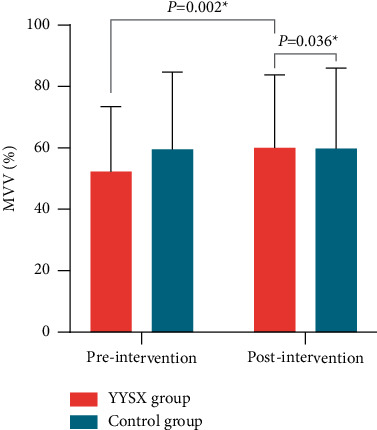
Comparison of maximum voluntary ventilation (MVV) in treatment and control groups between baseline and 2 weeks.

MVV, FVC, FEV1, and IC tested by PFT were used to assess lung function. The MVV was significantly higher in the YYSX group compared with the control group (*P* > 0.05). Furthermore, it improved after 2 weeks treatment in the YYSX group compared to the baseline (*P* > 0.05) (Figure 4). FVC and FEV1 were not significantly different between groups (Table 2). There was no significant difference between the two groups in IC at the 2-week point, but there was a tendency (*P*=0.071). FVC, FEV1, and IC were also found improved in the YYSX group compared to the baseline (*P* > 0.05).

**Figure 5:**
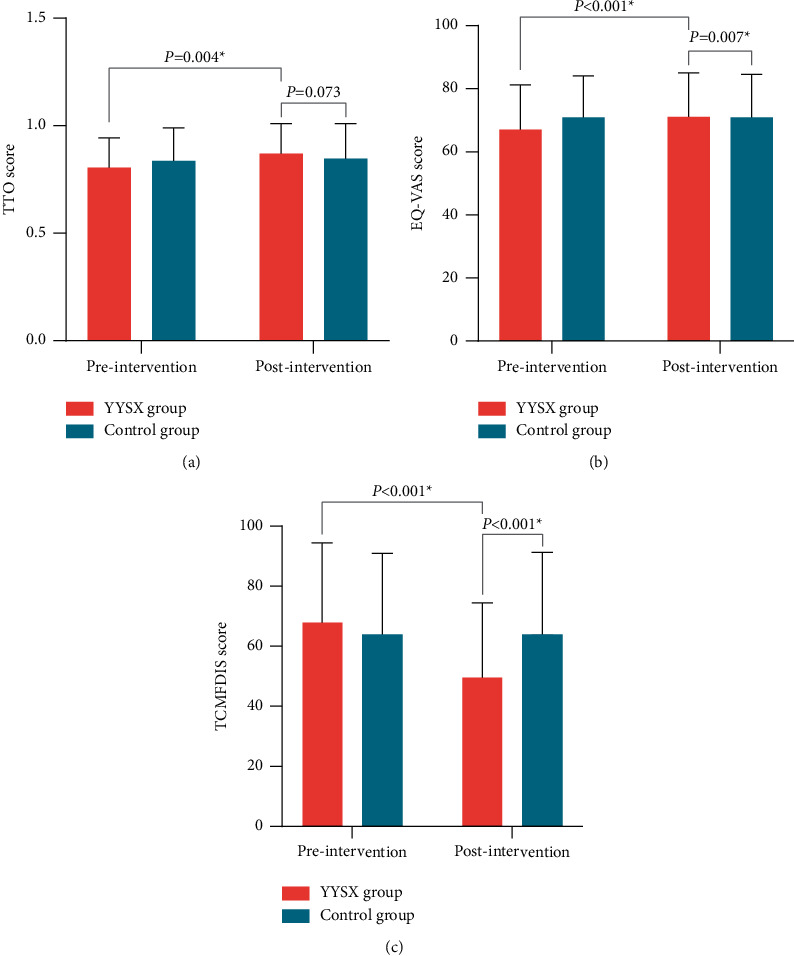
The effect of YYSX decoction in quality of life: a. comparison of the time trade-off (TTO) score in treatment and control groups between baseline and 2 weeks; b. Comparison of the EQ-visual analogue scale (EQ-VAS) score in treatment and control groups between baseline and 2 weeks; and c. comparison of the Traditional Chinese Medicine Four-Dimensional Diagnostic Information Scale (TCMFDIS) score in treatment and control groups between baseline and 2 weeks.

EQ-5D-5 L was used for measuring health-related quality of life, which generates a health utility index (UI) and a visual analogue scale (VAS) (range:1–100). The time trade-off (TTO) method was used to calculate the weighted sum of the UI values. There was no significant difference in the TTO score between the treatment group and control group after 2 weeks of treatment (*P* > 0.05). In the YYSX group, the TTO score was significantly higher than baseline after treatment (*P* > 0.05), whereas the change was similar to baseline in the control group. As for EQ-VAS score, there was significantly greater improvement in the YYSX group than in the control group (71.13 ± 13.95 vs. 70.94 ± 13.70, *P* < 0.007). Furthermore, the YYSX group had significantly higher EQ-VAS scores in the within-group comparison at 2 weeks compared to baseline (*P* < 0.05) (Figure 5).

The TCMFDIS score was compared between the YYSX and control groups, and a significant difference was observed between the two groups at 2 weeks (49.74 ± 24.73 vs. 64.16 ± 27.15, *P* < 0.001). The TCMFDIS score was also significantly higher compared with baseline in the treatment group (*P* < 0.05) (Figure 5).

There was no significant difference in the outpatient costs between the YYSX group and control group at baseline, 2–4-week, 4–8-weeks, and 8–12-week follow-ups (*P* > 0.05) (Table 3).

### 3.4. Safety

Among the 63 patients, one patient in the treatment group developed mild palpitations, which improved after stopping the YYSX decoction. No serious adverse events were observed. Although the average remained within the normal range, it is interesting to note that blood pressure (BP), including SBP and DBP, was significantly lower in the control group from baseline after 2 weeks (*P* > 0.05), but not in the YYSX group. Levels of serum sodium were lower in the YYSX group than in the control group after 2 weeks (*P* > 0.05). The results showed no clinically significant changes in other clinical laboratory tests (Table 4).

## 4. Discussion

Standard pharmacological therapies previously used for HFrEF, such as perindopril [13], irbesartan [14], candesartan [15], carvedilol [16], spironolactone [17], and sacubitril valsartan sodium [18], have failed to show a substantial benefit for HFpEF. As the first successful study in HFpEF, empagliflozin is currently the only drug that has proven to improve outcomes in patients with HFpEF [19]. Exercise intolerance is a cardinal symptom of HFpEF. Therefore, a drug that significantly improves exercise capacity and quality of life without compromising mortality and hospitalization offers hope for patients with HFpEF, like empagliflozin [19, 20].

This study shows that YYSX decoction was a potential and safe strategy for HFpEF patients because it can significantly improve exercise tolerance, quality of life, and cardiopulmonary function. Measurement of peak VO_2_ during CPET is recognized as the gold standard of exercise capacity. In this study, after two weeks of treatment, peak VO_2_ of the YYSX group rose by 7.6% (*P*=0.018), whereas that of the control group declined. MVV, EQ-VAS, and TCMFDIS scores of the YYSX group significantly improved after two weeks compared with the control group, suggesting the effect of YYSX decoction in improving cardiopulmonary function and quality of life. On the other hand, the two groups received additional health education to assist patients embrace heart-healthy habits such as salt and water restriction, weight control, physical activity, and cessation of smoking and alcohol, all of which may contribute to therapeutic benefits. It could explain why the therapies were effective in just two weeks. Improvements in exercise tolerance and quality of life may lead to a better long-term prognosis if treatment were sustained.

Previous studies including patients with HFpEF showed a slightly higher baseline peak VO_2_ level compared with our patient population [21]. On one hand, by using a cycle ergometer for peak exercise measurements may get a lower estimate of peak VO_2_ compared with treadmill testing. On the other hand, some patients may experience a subjective lack of effort due to fear of symptom triggering despite receiving a thorough explanation of the testing procedure. The cardiac ultrasound parameters LAVI, LVMI, and IVRT were improved in the YYSX group, but there was no significant difference between groups, as did BNP, which may be due to sample size limitations. Furthermore, the study's brief duration may not have been long enough to rule out such changes adequately if therapy was continued. It also implies that, to some extent, an increase in exercise tolerance is not exclusively connected to heart function. Previous studies suggested that peripheral mechanisms such as skeletal muscle abnormalities are also important mechanisms affecting exercise tolerance of HFpEF [22]. Therefore, we decided to carry out further animal experiments to explore the exact underlying mechanism of YYSX decoction in improving exercise tolerance in HFpEF.

Our findings were also consistent with previous clinical experience that YYSX decoction is a safe therapeutic approach in patients with HFpEF. Both groups were treated with conventional Western treatment (including antihypertensive drugs, diuretics, etc.), and there was no difference in BP at baseline. After 2 weeks, BP, including SBP and DBP, was significantly lower in the control group (rather than YYSX group) from baseline (*P* > 0.05), which indicated that conventional Western medicine treatment may increase the risk of low BP in people with HFpEF. The China Patient-Centred Evaluative Assessment of Cardiac Events Prospective Heart Failure Study has shown that among the hospitalized patients with HF, the lower admission SBP indicates the increased risk of all-cause mortality and HF readmission within one year [23]. Moreover, the level of serum sodium in the control group was slightly higher than that in the YYSX group after 2 weeks. Although this small change was still within the normal range, it was statistically significant (*P* > 0.05). The study has shown that in individuals with hypertensive HFpEF, a sodium-restricted diet was linked to improvements in ventricular diastolic function, arterial elastance, and ventricular-arterial coupling [24]. YYSX decoction may have a potential effect on preventing sodium retention.

In summary, these findings suggest that YYSX decoction should be considered a potential strategy for HFpEF patients, even more widely if not regarded as the standard regimen, at least as a complementary and alternative approach to conventional therapies. We believe the exploration of YYSX decoction would be valuable to the treatment of HFpEF. Future large, multicenter, prospective RCT studies with more scientific markers are warranted to provide convincing evidence on the effects of YYSX decoction, especially its long-term outcomes.

## 5. Limitations

We excluded patients in NYHA class IV who were unable to perform a symptom-limited exercise test to peak VO_2_ with an adequate effort, which may limit the extrapolation of the results to patients with worse heart function, and better results may be achieved in the clinical setting. The nature of the intervention makes it difficult to apply double-blindness, which may result in biased assessment. Finally, the follow-up period was relatively short, and whether a long-term follow-up would translate into the greater efficacy warrants further investigation.

## 6. Conclusion

The YYSX decoction combined with conventional Western treatment was superior to the conventional Western treatment alone in improving exercise tolerance, quality of life, and cardiopulmonary function of patients with HFpEF. The safety profile was shown to be satisfactory. Future multicenter, prospective RCT studies with more scientific markers are necessary to fully evaluate the efficacy of YYSX decoction in a larger patient population.

## Figures and Tables

**Table 1:** Baseline demographic and clinical characteristics.

Variables	Total (*n* = 63)	Treatment group (*n* = 31)	Control group (*n* = 31)	*P*
Age (yrs)	69.44 ± 6.04	69.32 ± 6.18	69.56 ± 5.99	0.876
Females (*n* %)	50 (79.37)	25 (80.65)	25 (80.65)	0.805
SBP (mmHg)	136.63 ± 16.60	133.23 ± 14.94	139.94 ± 17.68	0.109
DBP (mmHg)	78.16 ± 10.57	75.58 ± 10.26	80.66 ± 10.43	0.056
HR (bpm)	69.78 ± 13.63	69.16 ± 14.56	70.38 ± 12.87	0.727
Past medical history				
CHD (*n* %)	25 (39.68)	12 (38.71)	13 (40.63)	0.877
HTN (*n* %)	52 (82.54)	24 (77.42)	28 (87.50)	0.292
DM (*n* %)	16 (25.40)	7 (22.58)	9 (28.13)	0.613
Arrhythmia (*n* %)	29 (46.03)	14 (45.16)	15 (46.88)	0.891
HLD (*n* %)	30 (47.62)	14 (45.16)	16 (50.00)	0.701
NYHA functional classification				
Class II (*n* %)	49 (77.78)	22 (70.97)	27 (84.38)	0.201
Class III (*n* %)	14 (22.22)	9 (29.03)	5 (15.63)	0.201
Concomitant medications				
Aspirin (*n* %)	44 (69.84)	21 (67.74)	23 (71.88)	0.721
Beta-blockers (*n* %)	31 (49.21)	13 (41.94)	18 (56.25)	0.256
ACEI (*n*%)	2 (3.17)	2 (6.45)	0 (0.00)	0.147
ARB (*n* %)	28 (44.44)	12 (38.71)	16 (50.00)	0.367
CCB (*n* %)	31 (49.21)	13 (41.94)	18 (56.25)	0.256
Nitrate (*n*%)	16 (25.40)	5 (16.13)	11 (33.33)	0.096
Statin (*n* %)	35 (55.56)	15 (48.39)	20 (62.50)	0.260
MRA (*n* %)	6 (9.52)	4 (12.90)	2 (6.25)	0.372
Non‐MRA diuretics (*n* %)	13 (20.63)	6 (19.35)	7 (21.88)	0.805

Data are expressed by mean ± SD, median (interquartile range) or number (%) of patients.

**Table 4:** Comparison of the primary and secondary endpoints in the YYSX group and control group between baseline and 2 weeks.

Variables	YYSX group (*n* = 31)				Control group (*n* = 32)				
	Baseline	Week 2	Δ	*P* ^a^	Baseline	Week 2	Δ	*P* ^a^	*P* ^b^
Primary									
Peak VO_2_ (ml/kg/min)	11.19 ± 3.35	12.04 ± 3.41	0.84 (0.15 to 1.53)	0.018	11.56 ± 3.23	11.02 ± 3.33	−0.55 (−1.39 to 0.29)	0.192	0.013
Secondary									
CPET									
VO_2_@AT (ml/kg/min)	8.36 ± 2.23	8.75 ± 2.47	0.39 (−0.27 to 1.05)	0.232	8.85 ± 2.09	8.57 ± 2.63	0.28 (−0.87 to 0.32)	0.352	0.167
VE/VCO_2_ slope	31.34 ± 4.87	30.36 ± 5.66	−0.98 (−2.51 to 0.54)	0.198	30.29 ± 5.19	29.74 ± 6.17	−0.55 (−3.02 to 1.93)	0.656	0.980
Echocardiography									
LAVI (ml/m^2^)	25.80 (10.00)	24.11 (8.70)		0.039	23.00 (8.87)	23.95 (7.93)		0.362	0.483
LVMI (mg/m^2^)	107.41 (18.23)	102.68 (14.84)		0.034	103.40 (17.63)	105.79 (22.53)		0.412	0.945
IVRT (ms)	85 (26)	102 (42)		<0.001	98 (42.75)	103 (38)		0.876	0.752
E/e'	11.57 (7.34)	10.89 (4.71)		0.103	10.63 (3.20)	10.12 (3.58)		0.094	0.268
EDT (ms)	178 (53)	180 (69)		0.538	174.5 (64.5)	189.5 (66)		0.368	0.842
BNP (pg/ml)	72 (85.3)	51.4 (73.8)		<0.001	106.5 (81.68)	57.7 (94.93)		0.066	0.454
PFT									
MVV (%)	52.26 ± 21.19	60.06 ± 23.71	7.81 (3.11 to 12.50)	0.002	59.50 ± 25.23	59.81 ± 26.17	0.31 (−4.25 to 4.88)	0.890	0.036
FVC (%)	70.94 ± 18.26	75.32 ± 16.88	4.39 (−1.90 to 10.67)	0.164	72.08 ± 15.48	73.95 ± 15.93	1.88 (−3.48 to 7.23)	0.481	0.578
FEV1 (%)	75.61 ± 20.67	79.90 ± 17.72	4.29 (−1.36 to 9.95)	0.132	75.47 ± 18.64	76.64 ± 18.84	1.17 (−2.53 to 4.88)	0.524	0.295
IC	1.96 ± 0.79	2.11 ± 0.66	0.15 (−0.09 to 0.38)	0.219	1.98 ± 0.53	1.88 ± 0.65	−.0.09 (−0.27 to 0.08)	0.270	0.071
EQ-5D-5 L									
TTO score	0.806 ± 0.138	0.870 ± 0.141	0.06 (0.02 to 0.11)	0.004	0.837 ± 0.153	0.847 ± 0.163	0.01 (−0.03 to 0.05)	0.572	0.073
EQ-VAS score	67.03 ± 14.24	71.13 ± 13.95	4.10 (2.02 to 6.17)	<0.001	70.94 ± 13.16	70.94 ± 13.70	0.00 (−1.89 to 1.89)	1.000	0.007
TCMFDIS score	67.97 ± 26.49	49.74 ± 24.73	−18.23 (−24.16 to -12.29)	<0.001	63.94 ± 27.12	64.16 ± 27.15	0.22 (−5.38 to 5.82)	0.937	<0.001

Data are expressed by mean ± SD or median (interquartile range). *a* refers to the analysis of paired *T*-test. Comparison between baseline versus week 2. *b* refers to adjusted treatment differences estimates from an analysis of ANCOVA test, with intervention condition as the group factor and preintervention value as a covariate. Comparison between YYSX group versus control group in postintervention. Peak VO_2_, peak oxygen consumption; CPET, cardiopulmonary exercise test; VO_2_@AT, oxygen uptake at anaerobic threshold; VE/VCO_2_ slope, the minute ventilation/carbon dioxide production slope; LAVI, left atrial volume index; LVMI, left ventricular mass index; IVRT, isovolumic relaxation time; E/e', the ratio between early diastolic transmitral flow and mitral annular velocity; EDT, mitral E deceleration time; BNP, brain natriuretic peptide; PFT, pulmonary function test; MVV, maximum voluntary ventilation; FEV1, forced expiratory volume in 1 second; FVC, forced vital capacity; IC, inspiratory capacity; EQ-5D-5 L, European Quality of Life Five-Dimension Five-Level Scale; TTO, time trade-off; EA-VAS, EQ-visual analogue scale; TCMFDIS, Traditional Chinese Medicine Four-Dimensional Diagnostic Information Scale.

**Table 2:** Comparison of outpatient costs in the YYSX group and control group at baseline, 2–4 weeks, 4–8 weeks, and 8–12 weeks.

Variables	YYSX group (*n* = 31)	Control group (*n* = 32)	*P * ^*a*^
Baseline (yuan/day)	2.96 (7.48)	3.39 (3.87)	0.778
2–4 week (yuan/day)	3.20 (7.48)	3.64 (3.87)	0.385
4–8 week (yuan/day)	2.96 (8.02)	3.64 (3.85)	0.741
8–12 week (yuan/day)	2.96 (7.48)	3.85 (4.53)	0.880
*P* ^*b*^	0.331	0.967	

Data are expressed by median (IQR). *a* refers to the analysis of Mann–Whitney nonparametric rank test, comparison between YYSX group and control group. *b* refers to the analysis of Friedman test, comparison between baseline, 2–4 week, 4–8 week, and 8–12 week.

**Table 3:** Comparison of laboratory tests in the YYSX group and control group between baseline and 2 weeks.

Variables	YYSX group (n = 31)			Control group (n = 32)			
	Baseline	Week 2	*Pa*	Baseline	Week 2	*Pa*	*Pb*
SBP (mmHg)	133.23 ± 14.94	130.13 ± 15.64	0.218	139.94 ± 17.68	132.16 ± 18.21	0.007	0.638
DBP (mmHg)	75.58 ± 10.26	73.23 ± 8.72	0.141	81.28 ± 10.86	75.09 ± 9.05	<0.001	0.408
HR (bpm)	69.16 ± 14.56	70.61 ± 14.48	0.389	70.38 ± 12.87	69.34 ± 10.33	0.453	0.690
RBC (×10^9^/L)	4.29 ± 0.39	4.37 ± 0.46	0.250	4.37 ± 0.50	4.32 ± 0.48	0.121	0.662
WBC (×10^9^/L)	5.89 ± 1.30	5.99 ± 1.42	0.528	6.22 ± 1.39	6.13 ± 1.43	0.697	0.702
HGB (g/L)	129.65 ± 13.74	132.06 ± 14.91	0.193	132.88 ± 14.84	132.34 ± 14.25	0.552	0.938
PLT (×10^9^/L)	222.55 ± 48.39	224.35 ± 52.50	0.691	226.41 ± 67.05	227.69 ± 66.97	0.890	0.827
ALT (U/L)	22.61 ± 14.12	22.49 ± 13.55	0.906	20.08 ± 10.45	19.46 ± 8.71	0.541	0.294
Cr (*μ*mol/L)	35.35 ± 27.60	27.97 ± 20.91	0.025	51.58 ± 94.92	41.99 ± 70.16	0.293	0.290
BUN (mmol/L)	6.36 ± 2.94	6.53 ± 2.76	0.550	5.39 ± 1.45	5.71 ± 1.39	0.160	0.141
K^+^ (mmol/L)	4.24 ± 0.54	4.25 ± 0.49	0.838	4.09 ± 0.36	4.18 ± 0.31	0.207	0.460
Na^+^ (mmol/L)	142.11 ± 2.05	141.69 ± 2.71	0.178	142.71 ± 1.85	143.08 ± 1.56	0.252	0.015
Cl^−^ (mmol/L)	103.24 ± 4.25	102.86 ± 4.60	0.426	104.34 ± 2.90	104.59 ± 2.77	0.600	0.075

Data are expressed by mean ± SD. SBP, systolic blood pressure; DBP, diastolic blood pressure; HR, heart rate; RBC, red blood cell; WBC, white blood cell; HGB, hemoglobin; PLT, platelets; ALT, alanine aminotransferase; Cr, creatinine; BUN, blood urea nitrogen; Na^+^, Na^+^ concentrations; K^+^, K^+^ concentrations; Cl^−^, Cl^−^ concentrations. “*a*” refers to the analysis of paired T-test. Comparison between baseline versus week 2. “*b*” refers to adjusted treatment differences estimates from an analysis of ANCOVA test, with intervention condition as the group factor and preintervention value as a covariate. Comparison between YYSX group versus control group in postintervention.

## Data Availability

The data used to support the findings of this study are available from the corresponding author upon request.

## Abbreviations

ACEI: angiotensin-converting enzyme inhibitors
ALT: alanine transaminase
ARB: angiotensin II receptor blockers
BNP: B-type natriuretic peptide
BUN: blood urea nitrogen
CPET: cardiopulmonary exercise test
CPK: creatine phosphokinase
Cr: creatinine
CRF: case report form
DBP: diastolic blood pressure
E/e': the ratio of mitral peak velocity of early filling to early diastolic mitral annular velocity
EDT: mitral E deceleration time
EQ-5D-5L: European Quality of Life Five-Dimension Five-Level Scale
ESC: European Society of Cardiology
FEV1: forced expiratory volume in 1 second
FVC: forced vital capacity
HFpEF: heart failure with preserved ejection fraction
HGB: hemoglobin
HR: heart rate
IC: inspiratory capacity
IQR: interquartile range
LAVI: left atrial volume index
LVEF: left ventricular ejection fraction
LVMI: left ventricular mass index
MLHFQ: Minnesota Living with Heart Failure Questionnaire
MVV: maximum voluntary ventilation
NYHA: New York Heart Association
Peak VO_2_: the peak oxygen consumption
PFT: pulmonary function test
PLT: platelets
RBC: red blood cell
SD: standard deviation
TCM: traditional Chinese medicine
RCT: randomized controlled trial
SBP: systolic blood pressure
TCMFDIS: TCM Four-Dimensional Diagnostic Information Scale
VE/VCO_2_: the minute ventilation/carbon dioxide production
VO_2_@AT: oxygen uptake at anaerobic threshold
WBC: white blood cell
YYSX: Yangyin Shuxin.

## Supplementary Materials

Supplementary Materials Table S2. The CONSORT checklist.

## References

[1] Owan T. E., Hodge D. O., Herges R. M., Jacobsen S. J., Roger V. L., Redfield M. M. (2006). Trends in prevalence and outcome of heart failure with preserved ejection fraction. *New England Journal of Medicine*.

[2] Kitzman D. W., Little W. C., Brubaker P. H. (2002). Pathophysiological characterization of isolated diastolic heart failure in comparison to systolic heart failure. *JAMA*.

[3] Redfield M. M., Borlaug B. A. (2021). Quality of life and exercise ability in heart failure with preserved ejection fraction: No time for therapeutic complacency. *JAMA*.

[4] Wang J., Yang R., Zhang F. (2018). The effect of Chinese herbal medicine on quality of life and exercise tolerance in heart failure with preserved ejection fraction: a systematic review and meta-analysis of randomized controlled trials. *Frontiers in Physiology*.

[5] Tang Q. M., Zhao Z. Q., Wang X. L., Bi Y. F., Hou Y. Z. (2017). Syndrome differentiation and treatment of heart failure with normal ejection fraction. *Journal of Traditional Chinese Medicine*.

[6] Zhao Z. Q., Wang X. L., Zhang P. (2018). Yao HQ effect of Yangyin Shuxin decoction on quality of life of patients with heart failure with normal ejection fraction. *Journal of Traditional Chinese Medicine*.

[7] Malfatto G., Branzi G., Giglio A. (2012). Diastolic dysfunction and abnormal exercise ventilation predict adverse outcome in elderly patients with chronic systolic heart failure. *European Journal of Preventive Cardiology*.

[8] Guazzi M., Myers J., Peberdy M. A., Bensimhon D., Chase P., Arena R. (2010). Cardiopulmonary exercise testing variables reflect the degree of diastolic dysfunction in patients with heart failure-normal ejection fraction. *Journal of Cardiopulmonary Rehabilitation and Prevention*.

[9] Malfatto G., Cuoccio P., Bizzi C. (2009). [What underlies NYHA class in elderly patients with heart failure? Role of diastolic dysfunction, functional mitral regurgitation and abnormal ventilation during exercise]. *Monaldi Archives for Chest Disease*.

[10] Zhao Z., Wang X., Wang S. (2020). Research based on the core pathogenesis in the treatment according to traditional Chinese medicine syndrome differentiation for heart failure with normal ejection fraction. *Medicine (Baltimore)*.

[11] Kosmala W., Holland D. J., Rojek A., Wright L., Przewlocka-Kosmala M., Marwick T. H. (2013). Effect of If-channel inhibition on hemodynamic status and exercise tolerance in heart failure with preserved ejection fraction: a randomized trial. *Journal of the American College of Cardiology*.

[12] Fergusson D., Aaron S. D., Guyatt G., Hebert P. (2002). Post-randomisation exclusions: the intention to treat principle and excluding patients from analysis. *BMJ*.

[13] Cleland J. G., Tendera M., Adamus J. (2006). The perindopril in elderly people with chronic heart failure (PEP-CHF) study. *European Heart Journal*.

[14] Massie B. M., Carson P. E., McMurray J. J. (2008). Irbesartan in patients with heart failure and preserved ejection fraction. *New England Journal of Medicine*.

[15] Yusuf S., Pfeffer M. A., Swedberg K. (2003). Effects of candesartan in patients with chronic heart failure and preserved left-ventricular ejection fraction: the CHARM-Preserved Trial. *The Lancet*.

[16] Yamamoto K., Origasa H., Hori M., Investigators J.-D. (2013). Effects of carvedilol on heart failure with preserved ejection fraction: the Japanese Diastolic Heart Failure Study (J-DHF). *European Journal of Heart Failure*.

[17] Pfeffer M. A., Claggett B., Assmann S. F. (2015). Regional variation in patients and outcomes in the treatment of preserved cardiac function heart failure with an aldosterone antagonist (TOPCAT) trial. *Circulation*.

[18] Chandra A., Vaduganathan M., Lewis E. F. (2019). Ge J health-related quality of life in heart failure with preserved ejection fraction: the PARAGON-HF trial. *Journal of the American College of Cardiology: Heart Failure*.

[19] Anker S. D., Butler J., Filippatos G. (2021). Empagliflozin in heart failure with a preserved ejection fraction. *New England Journal of Medicine*.

[20] Khan M. S., Butler J., Greene S. J. (2020). Patient-reported outcomes for heart failure with preserved ejection fraction: conducting quality studies on quality of life. *European Journal of Heart Failure*.

[21] Udelson J. E., Lewis G. D., Shah S. J. (2020). Effect of praliciguat on peak rate of oxygen consumption in patients with heart failure with preserved ejection fraction: the CAPACITY HFpEF randomized clinical trial. *JAMA*.

[22] Kitzman D. W., Haykowsky M. J., Tomczak C. R. (2017). Making the case for skeletal muscle myopathy and its contribution to exercise intolerance in heart failure with preserved ejection fraction. *Circulation: Heart Failure*.

[23] Huang X. H., Liu J. M., Hu S. (2021). Systolic blood pressure at admission and long-term clinical outcomes in patients hospitalized for heart failure. *Esc Heart Failure*.

[24] Hummel S. L., Seymour E. M., Brook R. D. (2013). Low-sodium DASH diet improves diastolic function and ventricular-arterial coupling in hypertensive heart failure with preserved ejection fraction. *Circulation: Heart Failure*.

